# Traditional Chinese Medicine for adjuvant treatment of breast cancer: Taohong Siwu Decoction

**DOI:** 10.1186/s13020-021-00539-7

**Published:** 2021-12-02

**Authors:** Huajuan Jiang, Minmin Li, Kequn Du, Chuan Ma, Yanfen Cheng, Shengju Wang, Xin Nie, Chaomei Fu, Yao He

**Affiliations:** 1 State Key Laboratory of Southwestern Chinese Medicine Resources, Pharmacy College, Chengdu University of Traditional Chinese Medicine, 1166 Liutai Avenue, Wenjiang District, Chengdu, 611137 Sichuan China; 2 Guizhou Yibai Pharmaceutical Co. Ltd, Guiyang, 550008 Guizhou China

## Abstract

The high incidence of breast cancer is the greastest threat to women’ health all over the world. Among them, HER-2 positive breast cancer has the characteristics of high malignancy, easy recurrence and metastasis, and poor prognosis. Traditional Chinese medicine (TCM) has a rich theoretical basis and clinical application for breast cancer. TCM believes that blood stasis syndrome is one of the important pathogenesis of breast formation and development. Taohong Siwu Decoction (TSHWD) is based on the “First Prescription of Gynecology” Siwu Decoction. It is widely used in various blood stasis and blood deficiency syndromes, mainly in gynecological blood stasis. Clinical studies have found that THSWD can treat breast cancer by reducing blood vessel and lymphangiogenesis with auxiliary chemotherapy. In this study, we aim to explore the material basis and mechanism of THSWD in the treatment of HER-2 positive breast cancer through literature review and network pharmacology studies. Through a literature review of the traditional application, chemical composition of Chinese herbal medicine of THSWD, as well as its clinical reports and pharmacological research on breast cancer treatment. Meanwhile, we conducted “component-pathway-target” network through network pharmacology reveals the main material basis, possible targets and pathways of THSWD in inhibiting HER-2 positive breast cancer. Literature review and network pharmacology research results had predicted that, baicalein, kaempferol, caffeic acid, amygdalin, quercetin, ferulic acid, gallic acid, catalpol, hydroxysafflor yellow A, paeoniflorin in THSWD are the main effective chemical composition. THSWD regulates 386 protein targets and 166 pathways related to breast cancer. The molecular mechanism is mainly to improve the microenvironment of tumor cells, regulate the process of tumor cell EMT, and inhibit tumor cell proliferation and metastasis. This study revealed the mechanism of action of THSWD in the treatment of HER-2 positive breast cancer through literature review and network pharmacology studies, providing a scientific basis for clinical application.

## Background

Breast cancer has officially replaced lung cancer, becoming the cancer with the highest incidence rate in the world. The number of new breast cancers reached 2.26 million, and that of lung cancer was 2.2 million, it was released in the latest global cancer burden data for 2020 by the International Agency for Research on Cancer (IARC) of the World Health Organization. Breast cancer can be divided into Luminal (A and B), HER-2 positive and triple negative. Among them, breast cancer with HER-2 receptor overexpression has a high clinical incidence (15–20%), and the recurrence and metastasis rate is as high as 50% [1]. The HER-2 gene is a proto-oncogene, which amplification suggests that breast cancer is more malignant, more aggressive, and less responsive to standard chemotherapy and endocrine therapy. It is easier to relapse after treatment, and the survival period of patients is shortened by more than half [2, 3]. The birth of trastuzumab, a drug that inhibits HER-2 overexpression, has greatly improved the prognosis of patients with HER-2 positive breast cancer. However, the current research on the drug is still in its infancy, and the effective rate of single-drug application is low. It still needs to be further demonstrated in the clinical trial stage, and the possible toxic and side effects of its clinical application are not very clear. Therefore, the treatment of HER-2 positive breast cancer is still a major health concern worldwide [4–7].

Breast cancer is one of the 95 kinds of diseases which can be treated by Traditional Chinese medicine (TCM) effectively and recommended, which are recognized by the State Administration of Chinese Medicines. TCM believes that blood stasis and tumor are mutually cause and effect, and run through the progression of tumor. Blood stasis syndrome is one of the important pathogenesis of breast tumor formation and development. Modern clinical studies have shown that most of the blood metastases in breast cancer patients are accompanied by blood hypercoagulability. Especially in patients with HER-2 breast cancer, the proportion of blood stasis syndrome is significantly higher than that of other types of breast cancer. TCM believes that “blood stasis” is the key pathogenesis of breast cancer. “Stagnation of Qi and blood stasis” leads to poor blood circulation in the tumor and obstruction of tissue fluid drainage, resulting in a local hypoxic environment. Activating blood and removing blood stasis drugs can correct the hypoxia state of tumor tissue microenvironment by improving microcirculation [8, 9]. TCM prescriptions have the characteristics of multi-component, multi-target, and synergistic effect [10–12], which increasingly used to enhance the anticancer effect and control the side effects of radiotherapy and chemotherapy, such as Taohong Siwu Decoction (THSWD), Yanghe Decoction, Shugan Liangxue Decoction, and Guizhi Fuling Decoction are all clinically adjuvant treatments of breast cancer [13–15]. TCM for promoting blood circulation and removing blood stasis plays an important role in the treatment of breast cancer. Mainly because it can treat breast cancer by adjusting the microenvironment hypercoagulability, improving the hypoxic microenvironment and interstitial hypertension, inhibiting tumor cell metastasis, and inhibiting tumor angiogenesis [16, 17]. The researchers conducted a meta-analysis of the clinical efficacy of TCM combined with chemotherapy in the treatment of breast cancer, and found that Chinese herbal decoction for replenishing qi and nourishing blood and promoting blood circulation and removing blood stasis are frequently used. TCM combined with chemotherapy for breast cancer can reduce the incidence of severe myelosuppression, and it has greater advantages compared with chemotherapy alone in terms of the effective rate of objective curative effect and the effective rate of physical status score [18, 19].

THSWD is a prescriptions for breast cancer classification treatment included in the “Standards of the Chinese Society of Chinese Medicine/Guidelines for the Diagnosis and Treatment of Tumors in TCM”. THSWD is also a prescription included in the “List of Ancient Classic Prescriptions (First Batch)” issued by the National Administration of TCM and National Medical Produts Administration of the People's Republic of China. It consists of six herbs including *Rehmanniae Radix*, *Angelicae Sinensis Radix*, *Chuanxiong Rhizoma*, *Paeoniae Radix Alba*, *Persicae Semen* and *Carthami Flos*. It is based on Siwu Decoction, which is known as the “First Recipe in Gynecology” in TCM. It has the function of nourishing blood, promoting blood circulation and removing blood stasis. The Qing Dynasty medical book “Gynecology” (《妇科冰鉴》) recorded that THSWD was used to treat irregular menstruation caused by blood stasis, blood clots in menstrual blood, abdominal pain and bloating. The inheritance and innovation of modern physicians has widely used THSWD in the treatment of various systems throughout the body, and has been widely used in TCM clinics. THSWD is traditionally used in gynecology, the effective rate of treating primary dysmenorrhea is more than 90% [20], and it can be used for postpartum blood stasis [21], uterine bleeding [22], etc. It is widely used in internal medicine such as coronary heart disease [23, 24], ischemic stroke [25], etc.

THSWD can significantly improve the hypercoagulable microenvironment and microcirculation of local cancer tissues. It is mainly used to treat many gynecological diseases caused by blood deficiency and blood stasis, such as dysmenorrhea, breast hyperplasia, ovarian cysts, menopausal syndrome, etc. [20, 22]. Clinical studies have found that THSWD can treat breast cancer by reducing blood vessel and lymphangiogenesis with auxiliary chemotherapy. At the same time, THSWD can alleviate the side effects of bone marrow suppression caused by chemotherapy drugs [26]. In addition, we found that THSWD can inhibit the growth and metastasis of HER-2 positive breast cancer SK-BR-3 cells through in vitro cell test research, which mainly inhibits breast cancer metastasis by inhibiting the EMT process of tumor cells. It is clinically proven that THSWD, the traditional representative recipe for activating blood and removing blood stasis, can interfere with breast cancer and has clinical value. However, there are few relevant research on THSWD in the treatment of breast cancer. The clinical application, pharmacodynamics and molecular mechanism of THSWD in the treatment of breast cancer have not yet been systematically analyzed.

Network pharmacology combines the ideas of systems biology and multidirectional pharmacology. Network pharmacology analyzes the mechanism of action of the effective ingredients of drugs by constructing a complex network between “components-targets-pathways-disease”, and shifts from the traditional pharmacological research concept of finding a single target to the thinking of comprehensive network analysis. Network pharmacology can analyze the mechanism of action of TCM prescriptions as a whole, and can also independently describe the “active ingredients-targets-metabolic pathways” related to a specific disease. The “multi-ingredients” of TCM prescriptions and its “multi-pathway-multi-target” pharmacological effects have natural compatibility with network pharmacology [27–29].

Therefore, this article conducts a systematic review of the medicinal composition of THSWD, the traditional application of the medicinal materials, chemical composition, and the pharmacological research on the treatment of breast cancer, as well as the clinical research reports of THSWD in the treatment of breast cancer, which provides a scientific basis for THSWD to assist modern breast cancer treatment methods to interfere with breast cancer.

## Six TCMs in THSWD

### Traditional and modern clinical applications of six TCM herbs in THSWD

**Fig. 1:**
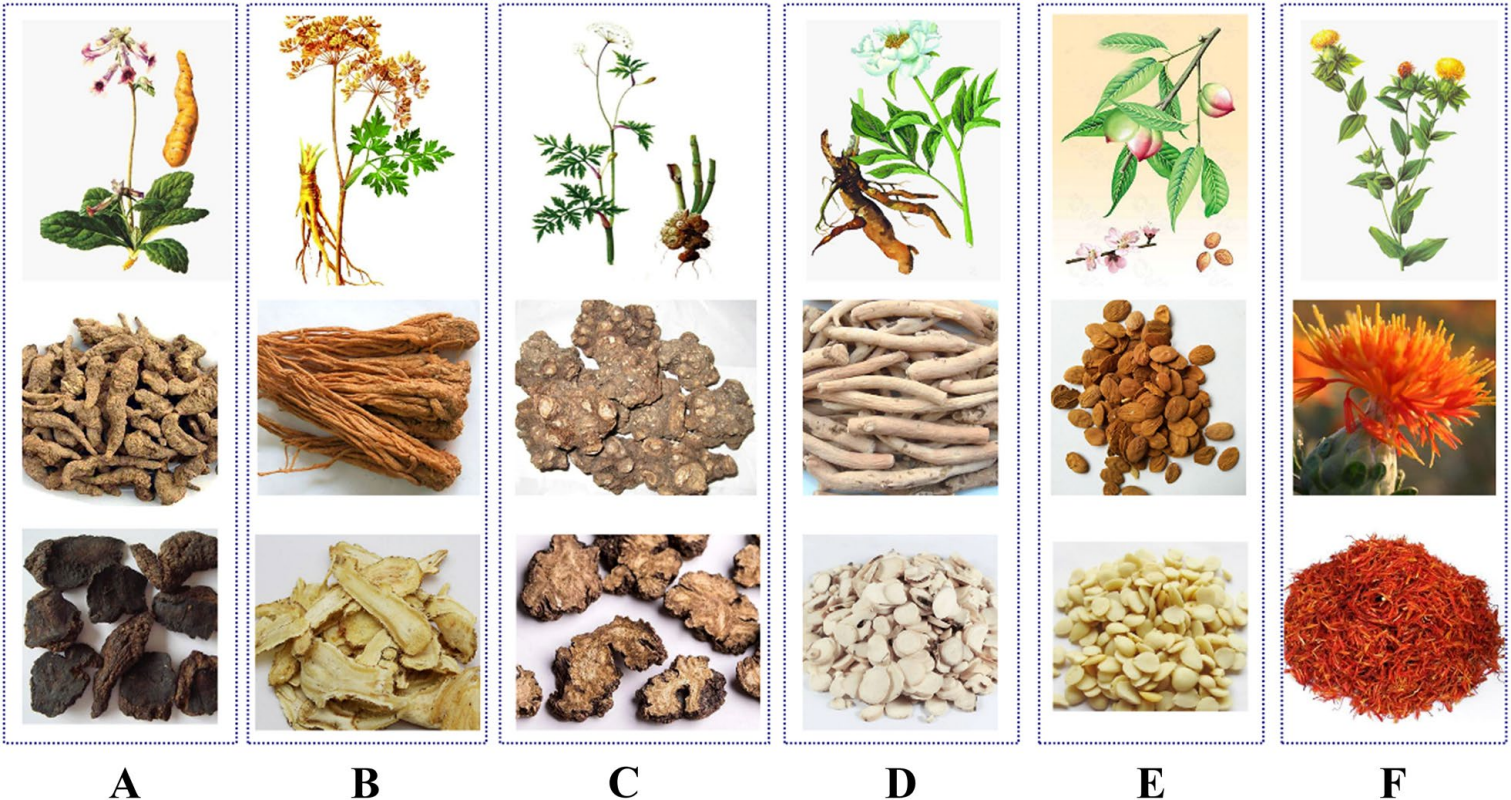
Above-ground parts, medicinal parts and decoction pieces of six TCM herbs in THSWD. **A** DH; **B** DG; **C** CX; **D** BS; **E** TR; **F** HH

THSWD is composed of six common traditional Chinese medicines: *Rehmanniae Radix* (Dihuang, DH), *Angelicae Sinensis Radix* (Danggui, DG), *Paeoniae Radix Alba* (Baishao, BS), *Chuanxiong Rhizoma* (Chuanxiong, CX), *Persicae Semen* (Taoren, TR), *Carthami Flos* (Honghua, HH). The detailed information of each medicinal material is shown in Table 1 and Fig. 1. We have systematically reviewed the origins, medicinal parts, properties and flavors of the six medicinal materials in THSWD, as well as their traditional applications, which will help to comprehensively understand the therapeutic effects of THSWD.

**Table 1:** Origin, flavor, meridian tropism, and traditional uses of six TCMs used in THSWD

TCM	REHMANNIAE RADIX (Dihuang, DH)	ANGELICAE SINENSIS RADIX (Danggui, DG)	PAEONIAE RADIX ALBA (Baishao, BS)	CHUANXIONG RHIZOMA (Chuanxiong, CX)	PERSICAE SEMEN (Taoren, TR)	CARTHAMI FLOS (Honghua, HH)
Origin	*Rehmannia glutinosa* Libosch	*Angelica sinensis*(Oliv.)Diels	*Paeonia lactiflora* Pall	*Ligusticum chuanxiong* Hort	*Prunus persica*(L.)Batsch or *Prunus davidiana*(Carr.)Franch	*Carthamus tinctorius* L
Medicinal parts	Root tuber	Root	Root	Rhizome	Seed	Flower
Producing area	Henan, Liaoning, Hebei, Shandong Province	Gansu, Yunnan, Sichuan Province	Sichuan, Zhejiang, Anhui Province	Sichuan, Yunnan, Guizhou Province	Sichuan, Shaanxi, Shandong, Hebei Province	Sichuan, Henan, Hunan, Xinjiang Province
Preparation	Bake slowly until about 80% dry	firework slowly smokes dry	After boiled in boiling water, remove skin and dry	Sun-dried	Sun-dried	Shade or sun-dried
Dosage	10–15 g	6–12 g	6–15 g	3–10 g	5–10 g	3–10 g
Flavor and meridian tropism	Sweet, cold; heart, liver, and kidney	Sweet, pungent, and warm; liver, heart, and spleen	Sweet, pungent, warm; liver and spleen	pungent, and warm; liver, gallbladder	Bitter, sweet, calm; heart, liver, large intestine meridian	Sweet, pungent; heart, liver
Efficacy	To clear heat and cool blood, nourish yin and promote body fluid	To Replenish blood; promote blood circulation; regulate menstruation and relieve pain; moisturize dryness and smooth intestines	To expel wind and dampness, detoxify and antispasmodic	Invigorate qi to relieve depression, eliminate wind and dampness, promote blood circulation and relieve pain	To promote blood circulation and remove blood stasis, moisten the intestines and laxative, relieve cough and relieve asthma	To activate blood circulation, remove blood stasis and relieve pain
Traditional uses	Used for heat into the camp blood, warm toxins and spots, vomiting blood, blemishes, and heat injury to the yin	Various syndromes of blood deficiency; irregular menstruation; amenorrhea; dysmenorrhea	Blood deficiency, irregular menstruation, abdominal pain, limb contraction pain, headache and dizziness	Wind-cold headache, dizziness, hypochondriac pain, abdominal pain, cold arthralgia and tendons, amenorrhea	Amenorrhea, dysmenorrhea, lumps, pulmonary carbuncle, intestinal carbuncle, fall and flutter injury, dry intestines, constipation, cough and wheezing	Treatment of amenorrhea, dysmenorrhea, dystocia, stillbirth
Main chemical composition	Catalpol, Rehmannia Glucoside D	Ferulic acid, ligustilide	Paeoniflorin, albiflorin, total glucosides of paeony	Ferulic acid, ligustilide, ligustrazine	Amygdalin	Hydroxysafflor yellow A, kaempferol, quercetin
Pharmacological action	Blood system, cardiovascular system, orthopedics, central nervous system and immune system	Anti-tumor, anti-oxidation, inhibition of angiogenesis, anti-inflammatory and pain-relieving	Protecting cardiovascular, anti-inflammatory, and anti-oxidation	Anti-tumor, anti-inflammatory, anti-oxidation, and protection of the nervous system	Cardio-cerebral vascular protection, inhibits atherosclerosis, it can anticoagulant, inhibit platelet aggregation and improve blood rheology	Anti-myocardial ischemia, regulating hemodynamics, anti-inflammatory, analgesic, anti-oxidation, and regulating immunity

DH has a long medicinal history and extensive pharmacological effects, and it is the monarch medicine of THSWD. It was first published in the *Shen Nong's Materia Medica* in the Qin and Han dynasties (221–220 BC). It records its sweet and cold taste and is mainly used in orthopedics. Tao Hongjing's *Collection of Materia Medica* records that DH can be used in gynecology. DH is now mainly produced in Henan, Liaoning, Hebei, and Shandong Province in China, and it is harvested in autumn. Its main effects are clearing heat and cooling blood, nourishing yin and promoting body fluid. Modern research has found that the types of chemical components of DH mainly include iridoids, ionones, phenethyl alcohol glycosides, triterpenes, flavonoids and sugars. Its main pharmacologically active ingredients are iridoid compound catalpol, rehmannia glycoside D, phenethyl alcohol compound verbascoside and so on [30, 31]. Modern pharmacological research shows that DH has significant effects on the human blood system, cardiovascular system, orthopedics, central nervous system and immune system [32–34].

DG is the dried root of *Angelica sinensis* (Oliv.) Diels, a plant of the Umbelliferae family. DG was first published in *Shen Nong's Materia Medica*, which tastes sweet, has a warm in nature and non-toxic. DG is mainly produced in Gansu Province and is also grown in Yunnan, Sichuan, Hubei Province. DG has a wide range of clinical applications. It has the function of tonifying blood, activating blood circulation, regulating menstruation and relieving pain, moistening the bowel, and treating irregular menstruation, dysmenorrhea, blood deficiency, and traumatic injuries. The chemical components of DG mainly include organic acids, phthalides, coumarins, etc., as well as some amino acids [35–37]. Among them, ferulic acid is the main active component of *Angelica sinensis* organic acid, which has many functions such as anti-inflammatory, anti-Alzheimer's disease, enriching blood and promoting blood circulation [38]. Ligustilide is the main component of the volatile oil of DG, which has various pharmacological effects such as anti-tumor, anti-oxidation, inhibition of angiogenesis, anti-inflammatory and pain-relieving [39].

BS is the dried root of *Paeonia tactiflora* Pall. of the Ranunculaceae family. It was first published in *Shen Nong's Materia Medica*. It tastes bitter, sour, and has a slightly cold in nature. Excavated in summer and autumn, it has the effects of nourishing blood, regulating menstruation, restraining yin and stopping sweating, softening liver and relieving pain, and suppressing liver yang. Indications of blood deficiency and chlorosis, irregular menstruation, abdominal pain, limb contracture, headache and dizziness. BS is a commonly used medicine for invigorating blood and regulating menstruation. Its chemical components mainly include monoterpenes, triterpenes, and flavonoids [40]. Among them, glycosides in BS, such as paeoniflorin and albiflorin, are its main active ingredients. Modern research shows that BS has the effects of protecting cardiovascular, anti-inflammatory, and anti-oxidation, and has been widely used in cardiovascular diseases, liver diseases, tumors and so on [10].

CX is the dried rhizome of *Ligusticum chuanxiong* Hort., a plant in the Umbelliferae family. It is warm in nature and pungent in taste. It was first published in *Shen Nong's Materia Medica*. It is mainly used for chest pain and heartache caused by heart stasis, flank pain caused by liver stagnation and qi stagnation, fall injury caused by blood stasis, irregular menstruation, various headaches and rheumatic arthralgia. Modern studies have shown that CX mainly contains phthalides, alkaloids, organic phenolic acids and their esters, polysaccharides and other ingredients. Its main pharmacologically active ingredients are ferulic acid, ligustilide, etc., which can protect the heart, brain, and blood vessels. Pharmacological effects such as anti-tumor, anti-inflammatory, anti-oxidation, and protection of the nervous system [41].

TR is a plant of the *Prunus persica *(L.) Batsch or *Prunus davidiana *(Carr.) Franch., and its dry mature seeds are used as medicine. Its nature and taste are bitter, sweet and calm. It has the effects of promoting blood circulation, removing blood stasis, moisturizing the bowel, relieving cough and asthma. It is used for amenorrhea, dysmenorrhea, pulmonary carbuncle, intestinal carbuncle, fall and flutter injury, dry intestinal constipation, cough and asthma. TR are mainly produced in Shandong, Sichuan, Shanxi, Hebei Province. There are many types of chemical components in TR, including volatile oils, cyanogenic glycosides, flavonoids, fatty acids, sterols, TR protein and other components [42]. Traditionally, amygdalin in cyanogenic glycosides is considered to be its characteristic component. Modern pharmacological studies have shown that TR has cardio-cerebral vascular protection, inhibits atherosclerosis, it can anticoagulant, inhibit platelet aggregation and improve blood rheology; at the same time, studies have shown that it can improve diabetic microangiopathy and increase peripheral nerves of the limbs, supply blood and nutrition, promote the metabolism of peripheral blood vessels and nerves and the recovery of damaged peripheral nerves [43].

HH is a dry tubular flower of *Carthamus tinctorius* L., which was first published in *Kaibao Materia Medica*. HH was originally produced in the upper reaches of the Nile in Egypt. It was expanded to Persia and then introduced into the Western Regions. HH has been introduced to my country after Zhang Qian’s envoy to the Western Regions. HH has a history of more than 2100 years and has been used as medicine for more than 1800 years. HH is pungent, slightly bitter, warm in nature. It is a good medicine for promoting blood circulation, dredging collaterals, removing blood stasis and relieving pain. Clinically, it is mainly used to treat high blood pressure, coronary heart disease, cerebral thrombosis and other cardiovascular and cerebrovascular diseases. The United States, Mexico, Canada, China, Ethiopia and some European countries, HH has large-scale planting in Xinjiang, Sichuan, Yunnan Province in China. HH contains flavonoids, alkaloids, polyacetylenes, spermidine, sterols, lignans, polysaccharides and other chemical components, of which kaempferol and hydroxysafflower yellow A are the main active ingredients [44]. Modern pharmacological studies have shown that hydroxysafflower yellow A has pharmacological effects such as anti-myocardial ischemia, regulating hemodynamics, anti-inflammatory, analgesic, anti-oxidation, and regulating immunity [45, 46].

The combination of the six medicinal materials in THSWD not only alleviates the coldness of the medicine, but also enhances the curative effect. It is widely used in various gynecological blood stasis syndromes, and modern clinical practice has also found that THSWD has a good clinical effect on blood stasis breast cancer.

## Clinical application of THSWD in treating breast cancer

TCM believes that stagnation of qi and blood stasis is the main pathogenesis of breast cancer. Most tumor patients have different degrees of blood stasis syndrome, and breast cancer patients have poor emotions, stagnated liver qi, resulting in poor blood flow, and long-term illness into the collaterals, resulting in blood stasis symptoms. Modern clinical studies have shown that 45% of breast cancer patients undergoing blood metastasis after TCM syndrome differentiation, blood stasis, toxin internal resistance, and blood stasis are common in most breast cancer patients, especially in patients with HER-2 breast cancer. The proportion of syndromes is significantly higher than other types of breast cancer. TCM for promoting blood circulation and removing blood stasis can increase vascular activity, which accelerates the inactivation of thrombin, inhibits the adhesion and aggregation of platelets, and stimulates the release of anticoagulant and fibrinolytic substances from vascular endothelial cells to improve the local hypercoagulable state of tumor tissue.

THSWD is one of the representative prescriptions for promoting blood circulation and removing blood stasis. It is widely used clinically in many obstetrics and gynecology diseases such as blood stasis dysmenorrhea caused by blood deficiency and blood stasis syndrome, postpartum blood stasis syndrome, and osteoporosis. The “Chinese Society of TCM Standard Tumor TCM Diagnosis and Treatment Guidelines” also recommend the heat-clearing and detoxifying decoction Wuwei Xiaodu Yin and the THSWD as TCM formulas for the treatment of breast cancer. Clinically, THSWD is widely used in the treatment of blood stasis internal resistance breast cancer.

THSWD combined with neoadjuvant chemotherapy (CAF/CEF or TAC/TEC regimen) can inhibit tumor lymphatic vessels and tumor angiogenesis in breast cancer patients. Its mechanism of action is related to the inhibition of VEGF-C and LVD, VEGF-A and MVD expression [47–49]. THSWD combined with neoadjuvant chemotherapy in the treatment of blood stasis and internal resistance breast cancer has a total remission rate of 90%. At the same time, it can reduce the degree of white blood cell decline and nausea, vomiting, gastrointestinal reaction, hair loss, bone marrow suppression. THSWD has good value in improving the efficacy of chemotherapy, improving the clinical symptoms of patients and reducing the toxic and side effects of drugs [50, 51]. THSWD combined with chemotherapy drugs in the treatment of invasive breast cancer can promote tumor cell apoptosis by down-regulating Bcl-2 protein, up-regulating Bax protein, and reducing Bcl-2/Bax ratio. At the same time, THSWD is effective in treating upper limb swelling after breast cancer surgery, and the cure rate can reach 87.9% [52].

In conclusion, THSWD can significantly improve the local hypercoagulable microenvironment after breast cancer surgery, and improve microcirculation. THSWD combined with chemotherapy can effectively reduce serum VEGF levels and lymphatic vessel density in breast cancer patients, thereby blocking angiogenesis and inhibiting tumor growth and metastasis.

## Pharmacological effects of single medicinal materials or chemical components in THSWD in the treatment of breast cancer

The therapeutic effect of THSWD on breast cancer remains at the clinical level, and there are few reports on related pharmacological studies. The active ingredients in the six medicinal materials of THSWD, such as paeoniflorin, catalpol, amygdalin, kaempferol, quercetin, ferulic acid, etc., have been confirmed by many studies to have the ability to treat breast cancer. We found that they mainly use the following four ways Anti-breast cancer: (1) inhibit breast cancer cell growth; (2) induce breast cancer cell apoptosis; (3) inhibit breast cancer cell migration and invasion; (4) reduce chemotherapy resistance of breast cancer cells.

### Inhibit the growth of breast cancer cells

Paeoniflorin can inhibit the NOTCH-1 signaling pathway by inhibiting NOTCH-1 and HES-1 protein expression levels, thereby inhibiting the proliferation of MCF-7 breast cancer cells [53]. However, this study only conducted in vitro pharmacological studies and lacked in vivo pharmacodynamic evaluations. Amygdalin showed strong cytotoxic effects on MCF-7 cells (IC_50_ = 14.2 mg/ml) and SK-BR-3 cells (IC_50_ = 13.7 mg/ml). Amygdalin can increase Bax and decrease Bcl- 2 expression [54]. Catalpol up-regulates the expression of miR-146a and down-regulates the expression of MMP-16 to inhibit the proliferation of MCF-7 breast cancer cells and promote their apoptosis [55]. The n-butenyl naphthalide extracted from angelica can induce G2/M phase arrest [56]. Quercetin can effectively inhibit the proliferation of MDA-MB231 cells and MCF-7 cells, and can effectively inhibit the expression of Hsp27, Hsp70 and Hsp90 without inducing DNA damage [57]. Quercetin inhibits p-AKT/AKT in BALB/c nude mice inoculated with MCF-7, inhibits tumor growth and metastasis, inhibits glycolysis, and induces autophagy [58]. In addition, quercetin treatment promotes weaker malignant activities related to cancer stem cells by inhibiting the PI3K/Akt/mTOR signaling pathway [59]. Kaempferol significantly induces proliferation inhibition and apoptosis of ZR-75–30 and BT474 breast cancer cells, while reducing the expression of IQGAP3. The up-regulation of IQGAP3 inhibits cell apoptosis, increases the expression of p-ERK1/2 and Bcl-2, and reduces the expression of Bax protein [60].

### Induce apoptosis of breast cancer cells

Amygdalin has cytotoxic effects on estrogen receptor positive MCF7 cells, MDA-MB-231 and triple negative Hs578T breast cancer cells. Amygdalin induces apoptosis of Hs578T cells. Amygdalin down-regulates Bcl-2 and up-regulates Bax protein, caspase-3 and PARP. Amygdalin activates the pro-apoptotic signaling molecule p38 MAPK in Hs578T cells [61]. In addition, amygdalin can increase the expression of pro-apoptotic Bax protein and decrease the expression of anti-apoptotic Bcl-2 protein in HER-2 positive breast cancer SK-BR-3 cells. Amygdalin can increase the pro-apoptosis Bax protein and reducing the expression of anti-apoptotic Bcl-2 protein induce SK-BR-3 cell apoptosis and death [62]. Angelica polysaccharide can significantly affect the expression of PARP, Bax protein, Bcl-2, Bcl-xL and Apaf1 protein in T47D breast cancer cells, and may induce cell apoptosis through the CREB signaling pathway [63]. Ferulic acid treatment can reduce the viability of breast cancer cell line MDA-MB-231, increase apoptosis and inhibit metastatic potential. In addition, the anti-tumor activity of ferulic acid and its effect of inhibiting metastasis are regulated by the reversal of EMT [64]. Kaempferol helps induce G2/M phase arrest, induce apoptosis and DNA damage in MDA-MB-231 cells. Kaempferol increased the expression levels of gH2AX, cleaved caspase 9, cleaved caspase 3 and p-A TM [65].

### Inhibit the migration and invasion of breast cancer cells

**Fig. 2:**
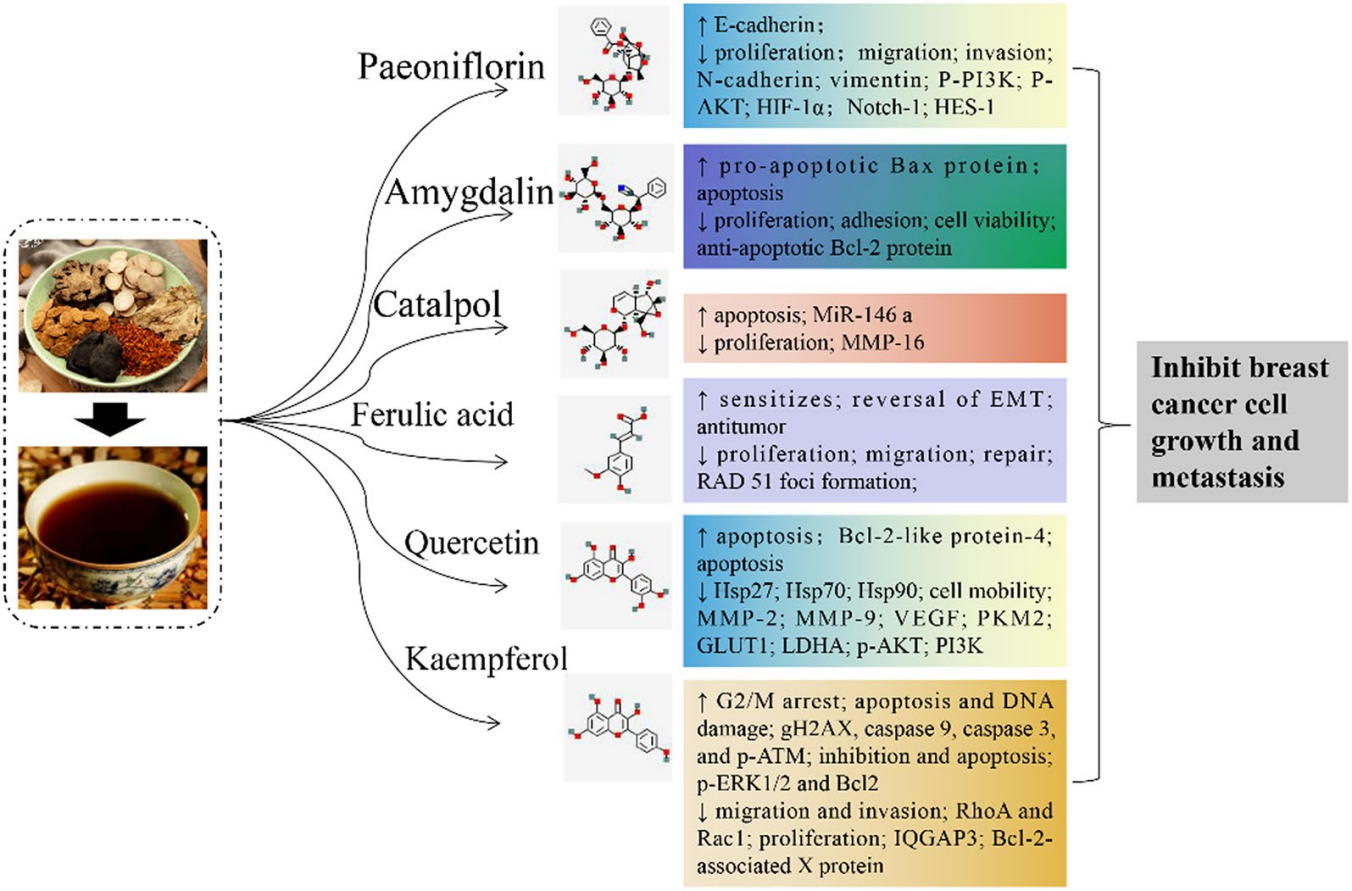
Summary of the pharmacological activities of the main active components in breast cancer

Paeoniflorin prevents the migration and invasion of MDA-MB-231 and MCF-7 breast cancer cells by inhibiting EMT under hypoxic conditions. Paeoniflorin can significantly reduce the increase in HIF-1α levels caused by hypoxia. In addition, paeoniflorin prevents the expression of phosphorylated PI3K and Akt in MDA-MB-231 cells induced by hypoxia [66]. Studies have also found that paeoniflorin inhibits the invasion of MDA-MB-231 and MCF-7 breast cancer cells by inhibiting the Notch1 signaling pathway [67]. N-butenyl naphthalene can inhibit the migration and invasion of breast cancer cells by inhibiting EMT [56]. Ligustrazine can inhibit the migration and invasion of MDA-MB-231 cells, by reducing the gene expression and activity of Akt, and increasing the activity of caspase-3 [68]. Quercetin inhibits the proliferation, self-renewal and invasiveness of breast cancer stem cells. It also reduces the expression levels of proteins related to tumorigenesis and cancer progression, such as acetaldehyde dehydrogenase 1A1, C-X-C chemokine receptor type 4, mucin 1, and epithelial cell adhesion molecules [69]. Kaempferol can inhibit the migration of SK-BR-3 and MCF-7 cells and the activation of RhoA [70]. The effect of the main active components of THSWD on breast cancer is shown in Fig. 2.

## Network pharmacological analysis of THSWD in treating HER-2 positive breast cancer

Modern clinical studies have shown that 45% of breast cancer patients undergoing blood metastasis after TCM syndrome differentiation, blood stasis, and internal resistance type. Most breast cancer patients have a blood hypercoagulable state, especially those with HER-2 breast cancer, the proportion of blood stasis syndrome is significantly higher than that of other types of breast cancer. THSWD is a representative prescription for promoting blood circulation and removing blood stasis. Therefore, the mechanism of THSWD in adjuvant treatment of HER-2 breast cancer was explored based on network pharmacology.

## Data preparation of network pharmacology

### Building a database of the main active components in THSWD

From the traditional Chinese Medicine System Pharmacology database (TCMSP, http://lsp.nwu.edu.cn/tcmspsearch.php), the traditional Chinese medicine potential target database (tcm-ptd, http://tcm.zju.edu.CN/PTD) and literature websites, including web of science (http://wokinfo.com/), PubMed (https://www.ncbi.nlm.nih.gov/) and China National Knowledge Infrastructure database (CNKI, https://www.cnki.net/), to explore the chemical constituents of six traditional Chinese medicines in THSWD. Oral bioavailability (OB) ≥ 30% and drug sample (DL) ≥ 0.18 were used as screening conditions, combined with serum pharmacochemical results and literature search, the candidate compounds were comprehensively screened for further analysis.

### Prediction of component targets

The main active components in THSWD were screened out and their structure diagrams were drawn with the function of “convert name to structure” under the structure option in Chembiodraw Ultra 12.0 software, and stored in MDL molfile (. Mol) format. Import the MDL molfile format file into OpenBabel software, and use its convert function to convert MDL molfile format into SDF MDL mol format file. Upload the SDF format structure file of ingredients to Swiss target prediction (http://www.swisstargetprediction.ch/) Server, reverse pharmacophore matching method was used to predict and analyze potential drug targets with “Homo sapiens” as screening criteria. Due to the nonstandard naming of drug targets, we used the UniProtKB search function in UniProt database (http://www.uniprot.org/). By inputting the protein name and defining the species as “human”, all the retrieved proteins were corrected to their official names. The protein information related to the active components was obtained through the database retrieval and transformation.

### Prediction of HER2-positive breast cancer targets

Through Genecards (http://www.genecards.org) and OMIM database (http://omim.org/) search for the keywords “HER2 positive breast cancer”, “luminal B HER2 positive breast cancer”, “HER2 overexpression breast cancer” to predict and collect HER-2 positive breast cancer related targets.

### Construction and analysis of protein protein interaction network

The potential targets of THSWD for the treatment of HER-2 positive breast cancer were obtained by intersection of component related targets and HER-2 positive breast cancer related targets. The data of potential targets of THSWD were imported into STRING 11.0 software (http://string-db.org) and the species was limited to “Homo sapiens”. The PPI network of protein interaction of THSWD was constructed by selecting the data with confidence higher than 0.9. The core genes of the network were found according to the number of genes and adjacent genes in the network.

### Enrichment analysis of GO and KEGG pathways

David bioinformatics resources 6.8 (https://david.ncifcrf.gov/) was used to analyze the enrichment of core target and PPI core gene in GO ( geneontology) biological process. KEGG (Kyoto Encyclopedia of Genes and Genomes, http://www.kegg.jp/) was used to annotate KEGG pathway of target, and significant pathways with P value ≤ 0.05 were selected for cluster analysis.

### Construction of component-target-pathway network

The screened components, target and pathway files were input into Cytoscape 3.7.2 to construct the “components-target-pathway” network diagram of THSWD. In the network, components, targets and pathway are represented by nodes, and the interaction between two nodes is represented by edges.

## Network pharmacology results

### Predicting the active ingredients of THSWD

Through ADME screening, 48 potential compounds in THSWD were identified (OB ≥ 30%, DL ≥ 0.18, caco-2 ≥ 0.4). In addition, through literature search, the screening added 14 potential active ingredients for the treatment of breast cancer. A total of 62 active compounds were selected from THSWD (Table.2).

**Table 2:** Active ingredients and parameters of THSWD

Pubchem CID	Abbreviation	Compound	OB (%)	DL	Herb
64971	BS1	Mairin	55.38	0.78	BS
442534	BS2	Paeoniflorin	53.87	− 1.47	BS
11092	BS3	Paeonol	28.79	0.04	BS
338	BS4	Salicylic acid	32.13	0.03	BS
370	BS5	Gallic acid	31.69	0.04	BS
9064	BS6	(+)-catechin	54.83	0.24	BS
70698143	BS7	Paeoniflorgenone	87.59	0.37	BS
9,841735	BS8	Palbinone	43.56	0.53	BS
5282184	CX1	Mandenol	42	0.19	CX
161748	CX2	Myricanone	40.6	0.51	CX
160179	CX3	Perlolyrine	65.95	0.27	CX
91726743	CX4	Senkyunone	47.66	0.24	CX
10873344	CX5	Wallichilide	42.31	0.71	CX
3085257	CX6	Senkyunolide A	26.56	0.07	CX
135398658	CX7	FA	68.96	0.71	CX
222284	B1	Sitosterol	36.91	0.75	CX; BS; DH
5319022	A1	(Z)-Ligustilide	53.72	0.07	CX; DG
689043	A2	Caffeic acid	25.76	0.05	CX; DG
1548883	A3	FERULIC ACID (CIS)	54.97	0.06	CX; DG
237332	SD1	5-Hydroxymethylfurfural	45.07	0.02	DH
5281605	HH1	Baicalein	33.52	0.21	HH
5280489	HH2	Beta-carotene	37.18	0.58	HH
5597	HH3	CLR	37.87	0.68	HH
5281238	HH4	Flavoxanthin	60.41	0.56	HH
261166	HH5	Lignan	43.32	0.65	HH
161739	HH6	Lupeol-palmitate	33.98	0.32	HH
5280784	HH7	Phytoene	39.56	0.5	HH
643672	HH8	Phytofluene	43.18	0.5	HH
457801	HH9	Poriferast-5-en-3beta-ol	36.91	0.75	HH
5281555	HH10	Pyrethrin II	48.36	0.35	HH
6443665	HH11	Hydroxysafflor yellow A	3.53	0.68	HH
5280445	HH12	Luteolin	36.16	0.25	HH
10237057	HH13	4-[(*E*)-4-(3,5-dimethoxy-4-oxo-1-cyclohexa-2,5-dienylidene but-2-enylidene]-2,6-dimethoxycyclohexa-2,5-dien-1-one	48.47	0.36	HH
5,281638	HH14	6-hydroxykaempferol	62.13	0.27	HH
5281680	HH15	Quercetagetin	45.01	0.31	HH
21786815	HH16	7,8-dimethyl-1*H*-pyrimido[5,6-g] quinoxaline-2,4-dione	45.75	0.19	HH
64982	HH17	Baicalin	40.12	0.75	HH
5280863	C1	Kaempferol	41.88	0.26	HH; BS
5280794	D1	Stigmasterol	43.83	0.76	HH; DG; DH
5280343	E1	Quercetin	46.43	0.28	HH; TR
1794427	E2	Chlorogenic acid	11.93	0.33	HH; TR
5281800	SD2	Acteoside	2.94	− 1.89	DH
91520	SD3	Catalpol	5.07	− 1.72	DH
656516	TR1	Amygdalin	4.42	− 1.91	TR
173283	TR2	Campesterol	37.58	0.71	TR
25245018	TR3	GA120	84.85	0.45	TR
73299	TR4	Hederagenin	36.91	0.75	TR
92735	TR5	Populoside_qt	108.89	0.2	TR
9548595	TR6	Sitosterol alpha1	43.28	0.78	TR
91747858	TR7	2,3-didehydro GA77	88.08	0.53	TR
91747861	TR8	GA121-isolactone	72.7	0.54	TR
91747856	TR9	GA122-isolactone	88.11	0.54	TR
5460209	TR10	GA19	88.6	0.46	TR
5460372	TR11	Gibberellin A44	101.61	0.54	TR
12310181	TR12	GA54	64.21	0.53	TR
13071237	TR13	GA60	93.17	0.53	TR
14160551	TR14	GA63	65.54	0.54	TR
5460657	TR15	Gibberellin 7	73.8	0.5	TR
131752128	TR16	GA77	87.89	0.53	TR
131752584	TR17	GA87	68.85	0.57	TR
9945785	TR18	3-*O-p*-coumaroylquinic acid	37.63	0.29	TR
222284	F1	Beta-sitosterol	36.91	0.75	TR; HH; DG; BS

### Compound-target network construction

**Fig. 3:**
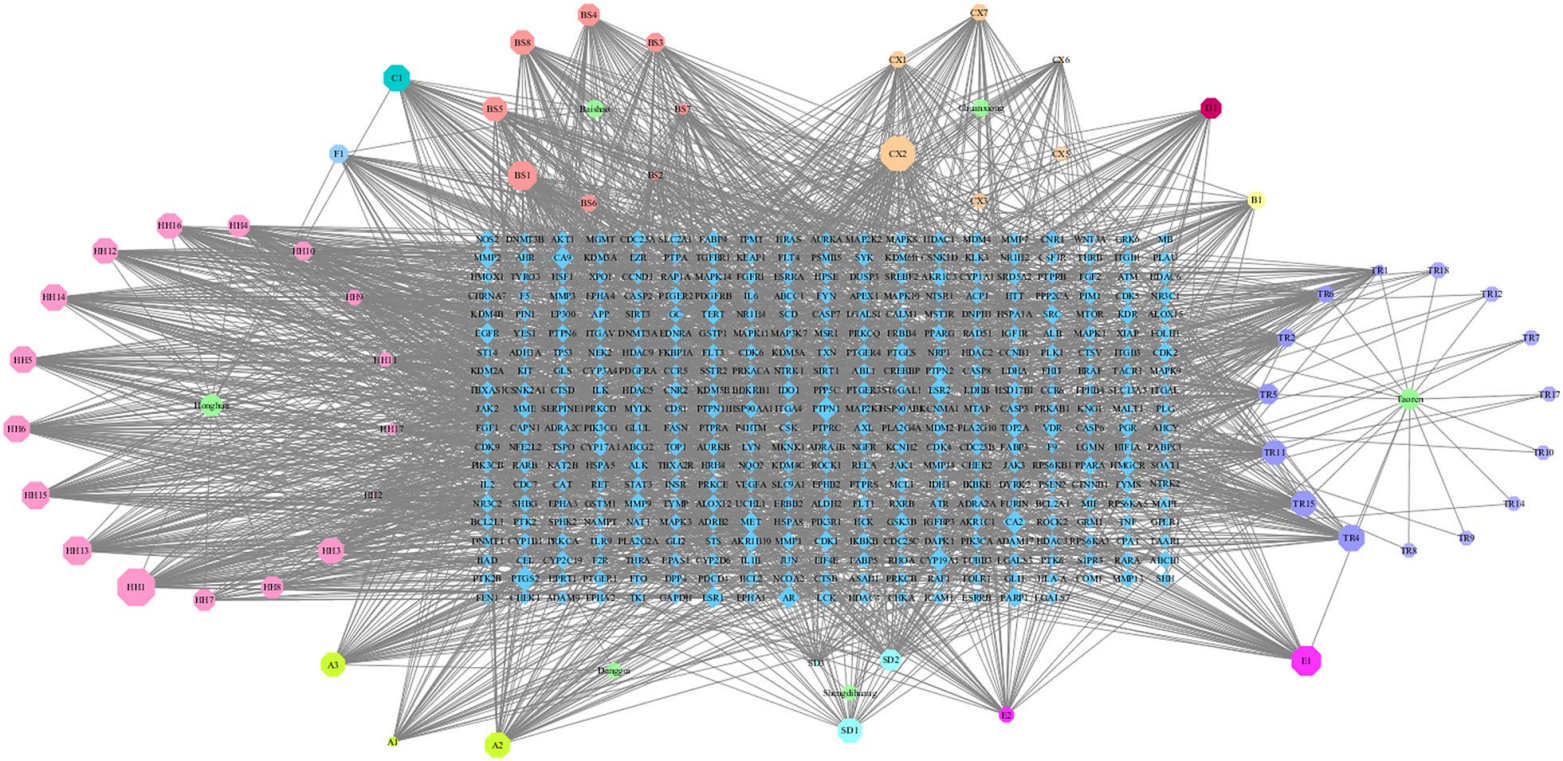
“Herbs-components-target”network

In this study, we used Cytoscape software to construct “herb-component-target” network model for exploring the potential mechanism by which THSWD treats HER-2 positive breast cancer. The relationship among the 58 active ingredients, their multiple targets, and their multiple pathways are shown in Fig. 3, which included 450 nodes (6 for herbs, 386 for proteins and 58 components). Among them, baicalein, kaempferol, caffeic acid, amygdalin, quercetin, ferulic acid, gallic acid, catalpol, hydroxysafflor yellow A, paeoniflorin correspond to more targets.

The blue diamond represents the target, the green circle represents the medicinal material, and the octagon next to each medicinal material represents the specific component of each medicinal material. The remaining scattered octagons represent the common components of some medicinal materials, and the size of the nodes is proportional to their degree value.

### Construction of PPI network and analysis of transcription factors

**Fig. 4:**
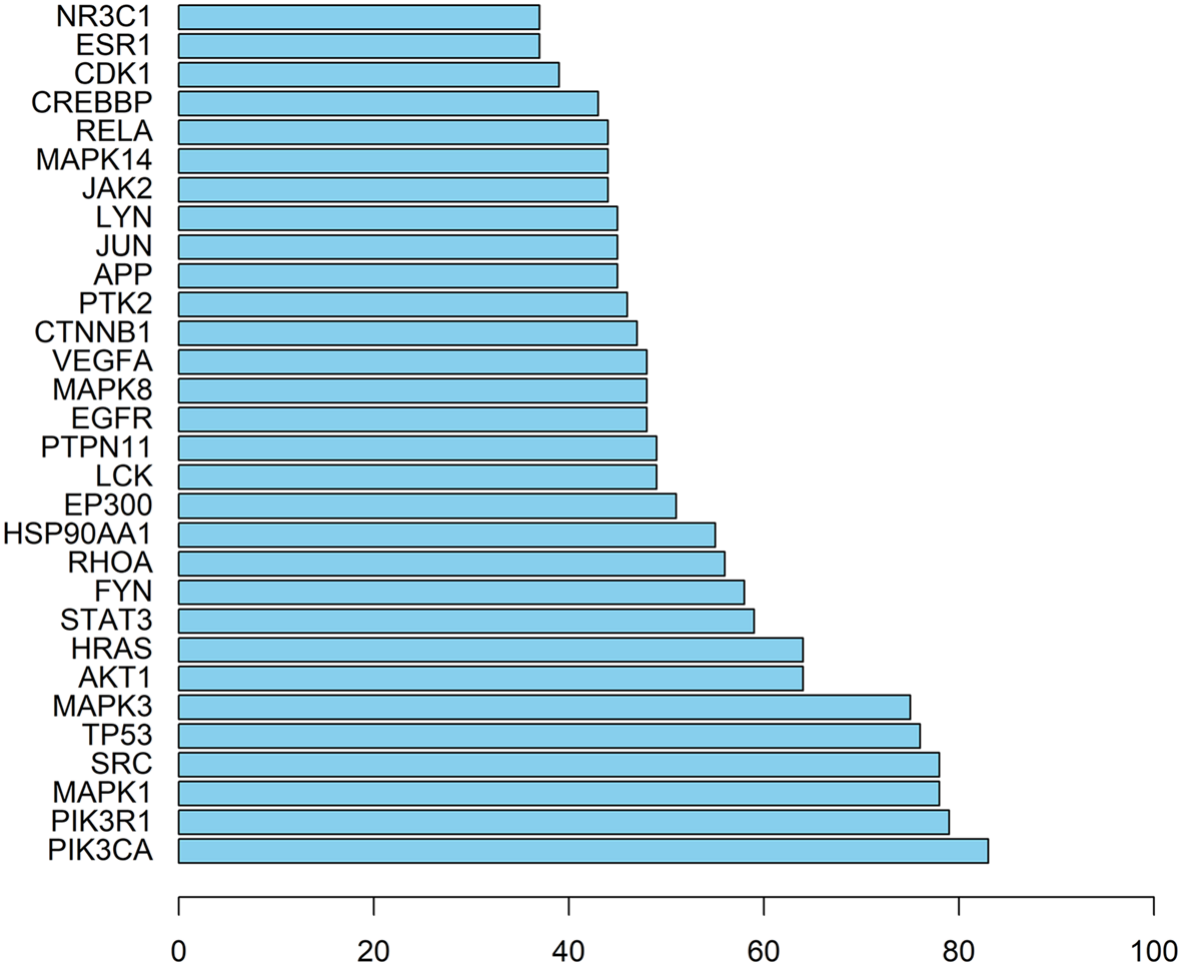
Histogram of core targets (top 30)

**Fig. 5:**
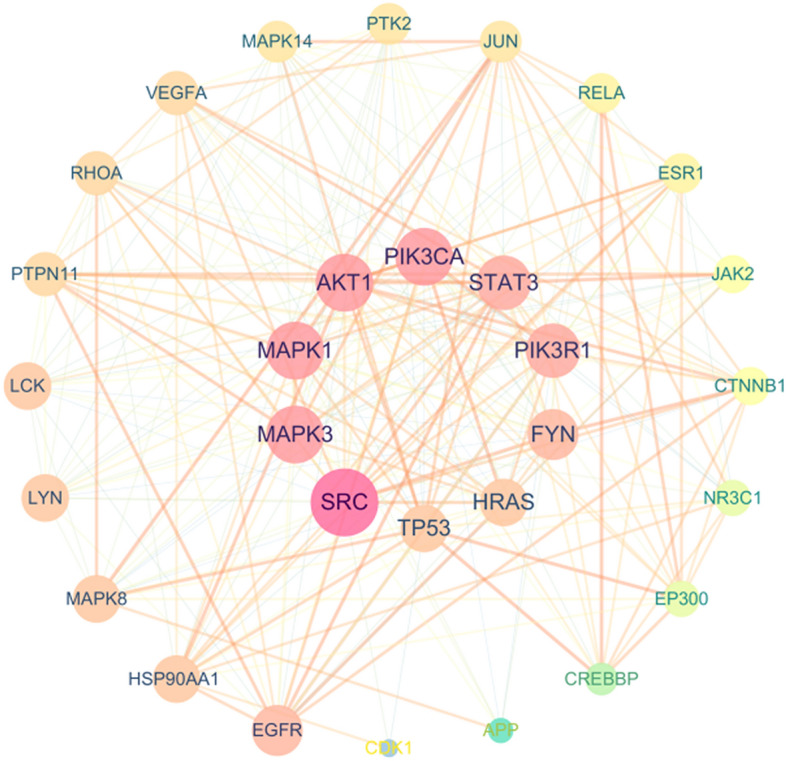
PPI Association Network

Enter the predicted 386 target proteins into the String database to obtain the PPI network, the result shows 346 nodes and 2696 edges. The top 30 targets with scores are shown in Fig. 4. Using Cytoscape 3.7.2 to draw the degree algorithm, the top 30 proteins were screened out in the form of a network diagram, and the results are shown in Fig. 5. The key targets include: phosphatidylinositol-4,5-Bisphosphate 3-Kinase Catalytic Subunit Alpha (PIK3CA, degree = 83); Phosphoinositide-3-Kinase Regulatory Subunit 1 (PIK3R1, degree = 79); Mitogen-Activated Protein Kinase 1 (MAPK1, degree = 78); SRC Proto-Oncogene, Non-Receptor Tyrosine Kinase (SRC, degree = 78); Tumor Protein P53 (TP53, degree = 76); Mitogen-Activated Protein Kinase 3 (MAPK3, degree = 75); AKT Serine/Threonine Kinase 1 (AKT1, degree = 64); HRas Proto-Oncogene, GTPase (HRAS, degree = 64); Signal Transducer And Activator Of Transcription 3 (STAT3, degree = 59); FYN Proto-Oncogene, Src Family Tyrosine Kinase (FYN, degree = 58).

The PPI correlation network diagram of the top 30 targets, the node size and color depth are proportional to their degree value.

### GO enrichment analysis

**Fig. 6:**
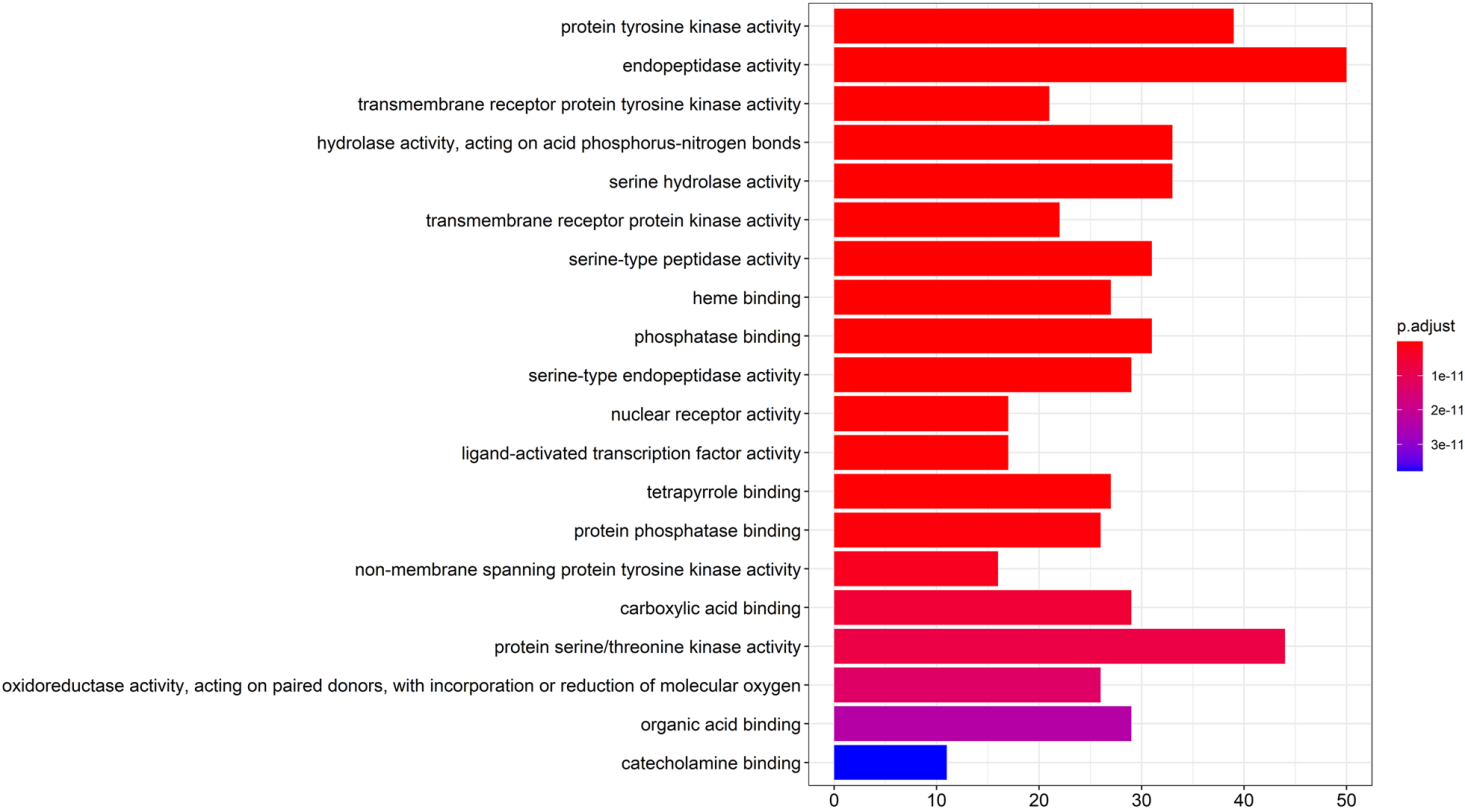
The gene ontology (GO) enrichment analysis for key targets

In the GOBP enrichment analysis, the GO enrichment analysis of 386 potential therapeutic targets was used to compare the enrichment degree of different genes in the GO term. The enrichment results of the first 20 biological processes are shown in Fig. 6. The results show that 386 targets are involved in various biological processes such as promoting tumor cell apoptosis and inhibiting tumor proliferation, immune regulation, blood circulation system regulation, and endocrine regulation.

### Pathway enrichment and compound-target-pathway network construction

**Fig. 7:**
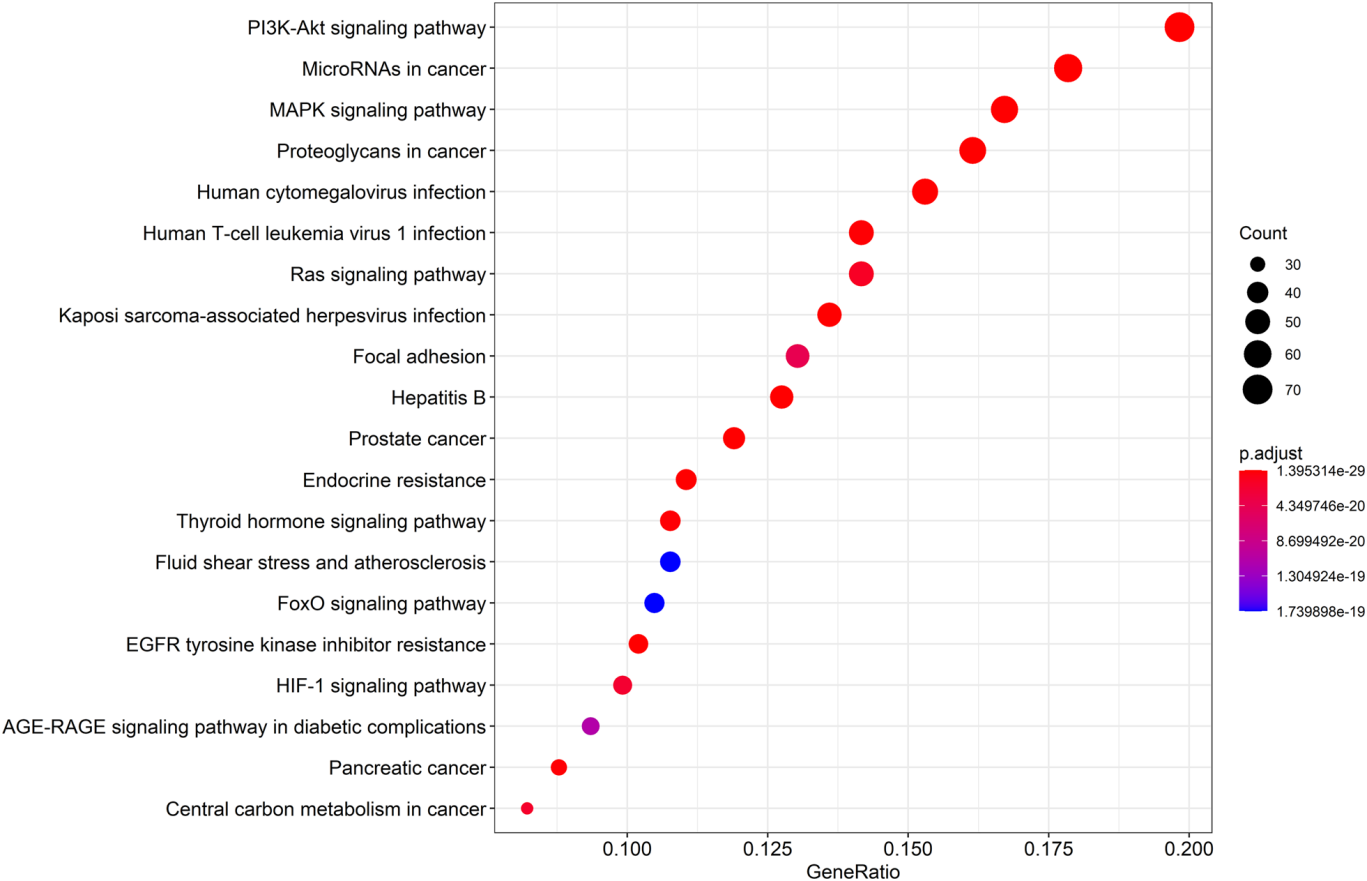
The KEGG pathway enrichment analysis of key targets

**Fig. 8:**
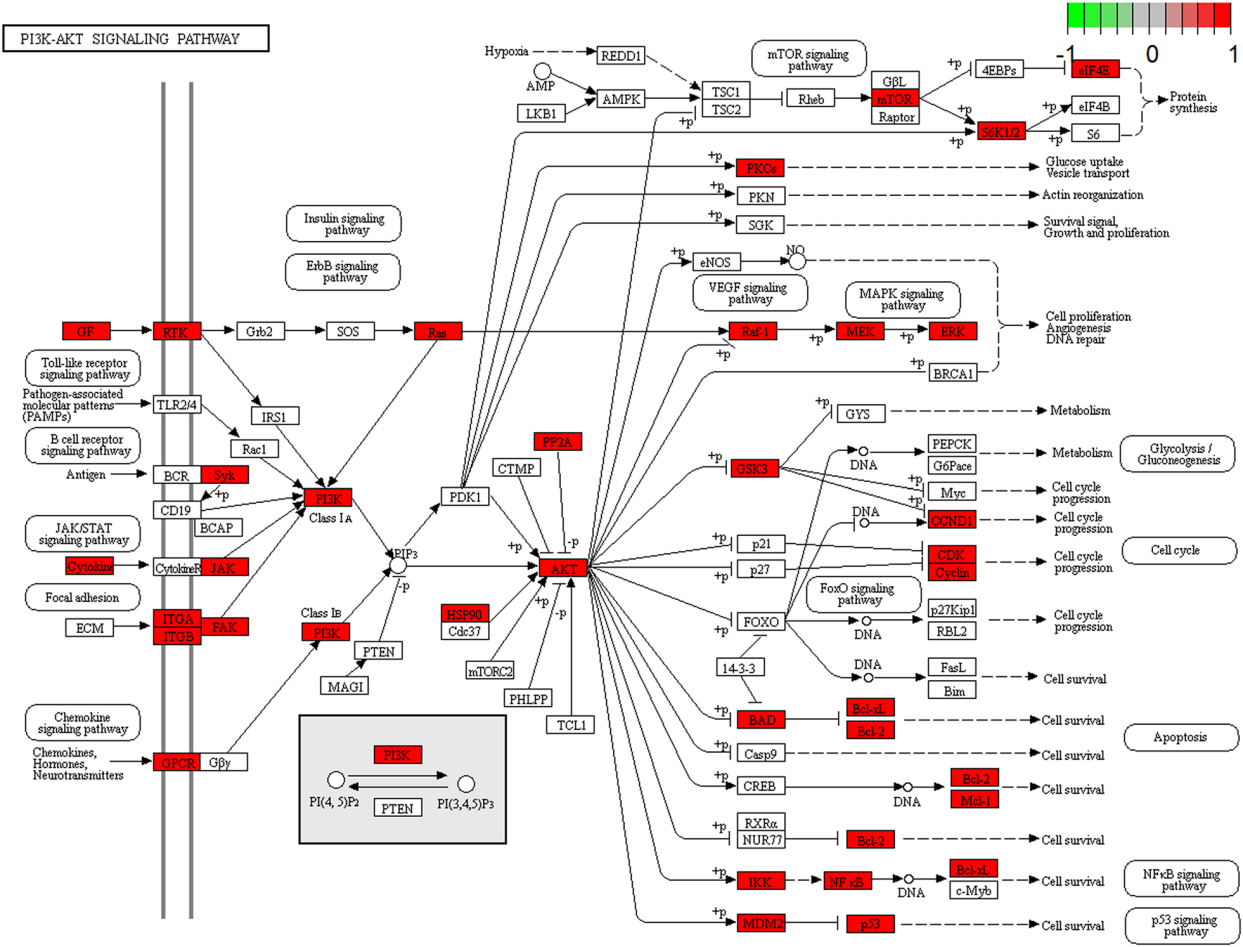
PI3K/AKT signal conduction diagram

**Fig. 9:**
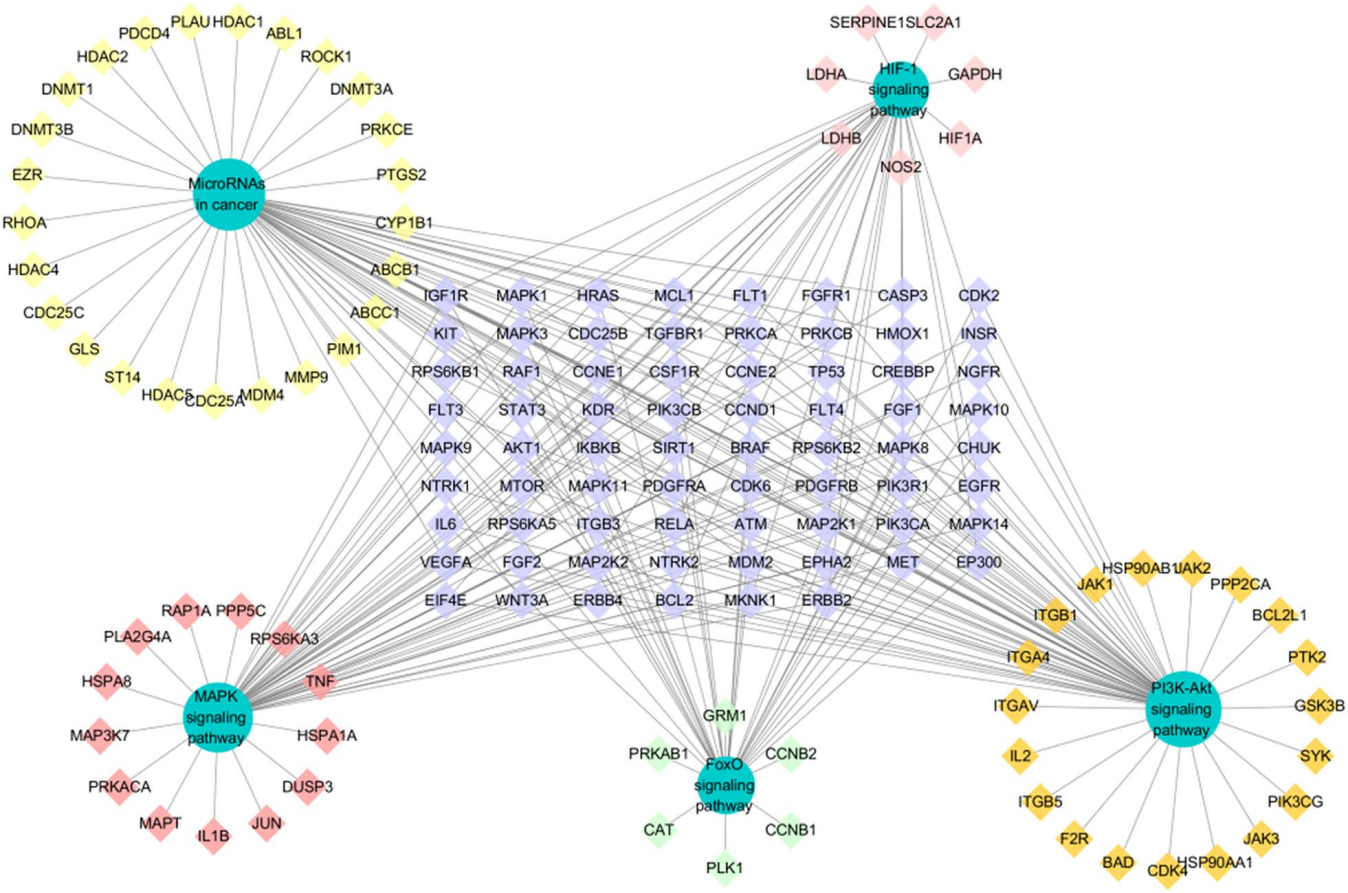
“Target-pathway”network diagram

In order to explore the potential pathway of THSWD in the treatment of HER-2 positive breast cancer, KEGG pathway enrichment was performed on 386 potential therapeutic targets. The top 20 significantly enriched pathways are shown in Fig. 7. Among these potential pathways, PI3K/AKT signaling is the most prominent pathway based on the number of genes, as shown in Fig. 8. Secondly, MAPK, MicroRNAs, FoxO, and HIF-1 signal pathway genes are enriched in large numbers, which are the key pathways for THSWD to treat HER-2 positive breast cancer, as shown in Fig. 9. There is a close synergy between key pathways, which can regulate the key biological processes of breast cancer occurrence and development, such as PI3K/AKT, MAPK signaling pathways are closely related to tumor cell proliferation and metastasis. The FoxO and HIF-1 signaling pathways can interfere with the tumor hypoxic microenvironment, and the MicroRNAs signaling pathway can regulate the EMT of tumor cells and intervene in the metastasis of tumor cells, etc.

The target marked in red in the figure is the target of the components in THSWD.

The green circle represents each channel, the diamonds around each channel represent its exclusive target, and the diamonds arranged in a rectangle represent the common target of each channel.

## Discussion

### THSWD is a typical representative of TCM for the treatment of breast cancer

Chinese herb and formulas for promoting blood circulation and removing blood stasis is a common treatment rule in TCM clinical treatment of tumor. Modern medicine also believes that cancer patients have abnormal blood hypercoagulability, and the formation of cancer thrombi is closely related to the abnormal blood clotting during tumor growth, proliferation, and metastasis. The mechanism of this abnormal hypercoagulable state is still more complicated, but in general it is believed to be related to the procoagulant directly produced by tumors and the procoagulant properties of normal cells activated by tumor cells. The method of activating blood and removing blood stasis can improve the hypercoagulable state of the blood of cancer patients, regulate the tumor microenvironment, inhibit the growth of microvessels, and improve the immune function of the body to inhibit tumor occurrence, development and outcome. It can improve the blood stasis syndrome of patients and improve the survival of patients quality, this is also the advantage of TCM in treating cancer [71–73].

THSWD is a classic prescription for promoting blood circulation and removing blood stasis. The clinical application of THSWD in breast cancer has also shown that THSWD can effectively correct abnormal blood rheology, reduce blood viscosity and improve microcirculation. After breast cancer surgery, the red blood cell aggregation is enhanced and the deformability is weakened, which can increase the microcirculation resistance, slow the blood flow rate, cause microcirculation disorders, and cause lymphedema of the upper limbs. THSWD can relieve lymphedema of the upper limbs after breast cancer surgery.

### Natural chemical components in THSWD inhibit breast cancer growth and metastasis

TCM prescriptions have the characteristics of multi-component, multi-target, multi-path comprehensive treatment of diseases. The natural chemical components of TCMs are the material basis for its curative effect. In the process of literature search, we found that the existing research of THSWD in the treatment of breast cancer is mostly focused on the clinical, and there are few literature reports that reveal its effects through pharmacological experiments. The active ingredients in THSWD, such as ferulic acid in DG and CX, paeoniflorin in BS, and catalpol in DH all exhibit the ability to inhibit breast cancer proliferation and metastasis. Therefore, this article conducts a systematic review of these documents in order to reveal its mechanism of action, and provide reference for the study of the mechanism of THSWD in the treatment of breast cancer and the study of its pharmacodynamic mechanism. In the process of literature review, we also found that the research on the relevant pharmacodynamic mechanism is mostly in vitro cell experiment research, and the overall pharmacodynamic experiment needs to be further carried out.

### Discussion on prediction results of network pharmacology

THSWD is a classic Chinese prescription for promoting blood circulation and nourishing blood. It has the theoretical basis of TCM for the treatment of breast cancer. Traditional Chinese medicine believes that the constitution of blood deficiency and blood stasis can easily lead to cancer, and the method of invigorating blood and nourishing blood has a long history of application in cancer treatment [72, 74]. For example, Shuangshen granules can inhibit lung cancer metastasis by promoting blood circulation and removing blood stasis [75], *Carthami Flos* is a commonly used traditional Chinese medicine for promoting blood circulation and removing blood stasis, which can prevent lung metastasis of breast cancer by inhibiting tumor cell invasion [73].

Many clinical studies have shown that THSWD has an adjuvant treatment effect on breast cancer, can relieve the side effects of bone marrow suppression caused by chemotherapy drugs, control the decline of white blood cells, and relieve nausea and vomiting. At the same time, THSWD can regulate the level of estrogen, regulate the patient's immune function and inhibit the tumor lymphangiogenesis to treat breast cancer.

### Screening the main active ingredients of THSWD in the treatment of breast cancer

As a commonly used clinical prescription for promoting blood circulation and removing blood stasis, THSWD has positive significance in the treatment of breast cancer. Studies have shown that THSWD can reduce the formation of blood vessels and lymph vessels in the microenvironment of breast cancer, improve the hypercoagulable microenvironment and microcirculation after breast cancer surgery, and achieve the purpose of inhibiting breast cancer growth and metastasis. The effective ingredients in the six herbs in THSWD on the prevention and treatment of breast cancer have also been studied by many researchers. Ferulic acid, the active ingredient in *Chuanxiong Rhizoma* and *Angelicae Sinensis Radix*, exerts anti-tumor activity and inhibits the metastasis of breast cancer cells by regulating EMT [64]. The active ingredient amygdalin in *Persicae Semen* has anticancer activity in vitro and in vivo on SK-BR-3 breast cancer cell line [62]. Catalpol, an active ingredient in *Rehmanniae Radix*, inhibits the proliferation of MCF-7 breast cancer cells and promotes apoptosis by up-regulating microRNA-146a and down-regulating the expression of matrix metalloproteinase-16 [55]. Paeoniflorin, the active ingredient in *Paeoniae Radix Alba*, inhibits the proliferation and invasion of breast cancer cells by inhibiting the Notch-1 signaling pathway [67]. At the same time, paeoniflorin can prevent hypoxia-induced EMT of human breast cancer cells [66]. The phytoestrogen kaempferol has a significant inhibitory effect on primary tumor growth and lung metastasis in mouse breast tumor models [76, 77]. Kaempferol can inhibit breast cancer cell proliferation, induce cell cycle arrest, apoptosis, DNA damage [65], and EMT related behavior [78]. Quercetin can inhibit glycolysis by autophagy mediated by Akt-mTOR pathway, thereby inhibiting breast cancer metastasis [58]. Quercetin can enhance the chemotherapy effect of adriamycin on human breast cancer cells and reduce its side effects [79].

At the same time, the research team used network pharmacology and found that baicalein, kaempferol, caffeic acid, amygdalin, quercetin, ferulic acid, gallic acid, catalpol, hydroxysafflor yellow A, paeoniflorin and other ingredients in THSWD play an important role in the treatment of breast cancer. The content of the ingredients in the decoction should not be ignored, these ingredients are also higher in THSWD. They are the material basis for THSWD to inhibit the growth and metastasis of breast cancer.

### Predict the mechanism of action of THSWD in the treatment of breast cancer based on “target-pathway”

THSWD improves the hypercoagulable state of the tumor microenvironment by accelerating the inactivation of thrombin, inhibiting the adhesion and aggregation of platelets, and stimulating the release of anticoagulant and fibrinolytic substances from vascular endothelial cells. THSWD combined with chemotherapy can effectively reduce serum VEGF levels and lymphatic vessel density in breast cancer patients, thereby blocking angiogenesis and inhibiting tumor growth and metastasis, but its mechanism of action is still unclear [80, 81]. Existing clinical studies have also shown that THSWD can inhibit angiogenesis and endothelial cell proliferation, reduce tumor microvessel density, prevent the binding of VEGF and its receptor, and down-regulate serum MVD and VEGF-A in patients after surgery [82, 83].

**Fig. 10:**
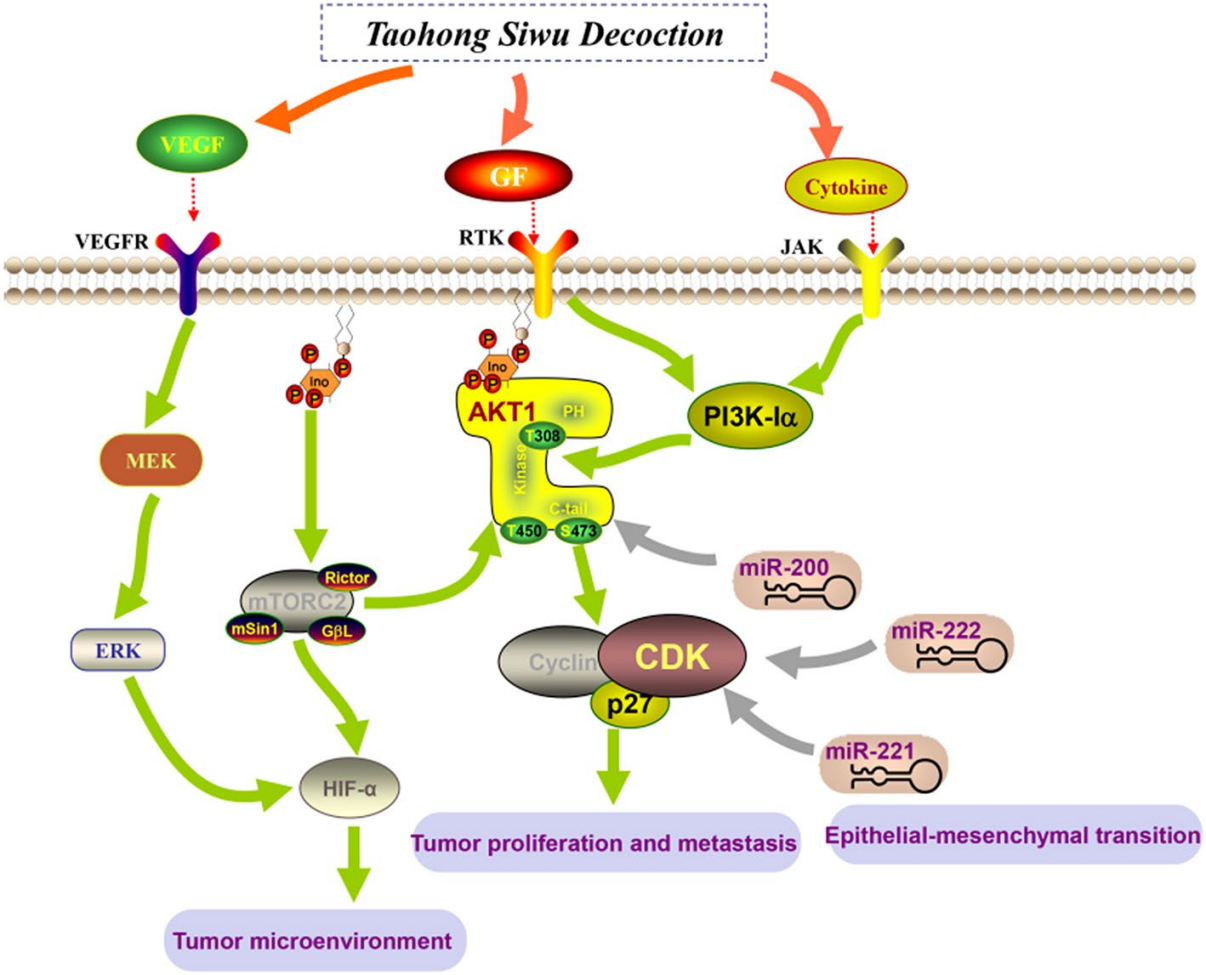
The key pathway diagram of THSWD in the treatment of HER-2 positive breast cancer

The results of the "component-target" network analysis showed that the efficacy of THSWD in the treatment of breast cancer is not only focused on promoting tumor cell apoptosis and inhibiting tumor proliferation (PIK3CA, PIK3R1, MAPK3, AKT1, JUN, STAT3) core targets, it also focuses on improving the tumor microenvironment, inhibiting angiogenesis (HIF, VEGFA), and immune regulation (IL6, IL1B, IL10) and other related targets. These targets are interconnected and can form more than 100 “target-pathway” network diagrams. The breast cancer-related pathways obtained from the analysis can be divided into three categories: inhibiting tumor cell proliferation and metastasis, intervening in the tumor hypoxic microenvironment, and regulating tumor cell EMT. Through data analysis and literature search, we can screen out three representative pathways respectively, namely PI3K/AKT signaling pathway, HIF-1 signaling pathway, and MicroRNAs signaling pathway in Fig. 10.

PI3K/AKT signaling pathway plays an important role in the occurrence of HER-2 positive breast cancer [84], it can promote breast cancer metastasis by inducing EMT [85]. At the same time, PIK3CA will promote the resistance of HER-2 targeted therapy [86], inhibitors of the PI3K/Akt/mTOR pathway are an important strategy to overcome resistance to anti-HER2 therapy [87, 88]. The detection of circulating microRNA through liquid biopsy is an important tool for the early diagnosis and prognosis of breast cancer. MicroRNA plays a key role in the occurrence and development of breast cancer. In the process of breast cancer, MicroRNA can regulate PI3K/AKT signaling pathway [89]. For example, the regulation of PI3K/Akt signal by MiR-204-5p can inhibit tumor metastasis and immune cell reprogramming in breast cancer [90]. MiR-129-5p can increase the enhancement of trastuzumab in the treatment of HER-2 positive breast cancer [91]. HIF-1 signaling pathway plays an indispensable role in tumor hypoxia microenvironment [92], in aggressively growing tumors, hypoxia induces HIF-1α expression and promotes angiogenesis [93, 94]. Existing studies have shown that hypoxia enhances the function of MicroRNAs following the COX-2/EP4/PI3K/Akt pathway [95]. Down-regulation of MiR-20b can activate HIF-1α and VEGFA to promote breast cancer migration and invasion [96]. At the same time, hypoxia can induce MiR-153 to fine-tune HIF1α/VEGFA in breast cancer angiogenesis [97], MiR-100 can inhibit in vitro angiogenesis by regulating the mTOR/HIF-1α/VEGF signal transduction axis in breast cancer cells [98].

## Conclusions and prospects

THSWD, as a classic prescription for promoting blood circulation and removing blood stasis, has achieved a good effect in clinical auxiliary treatment of blood stasis breast cancer. This article reviews the traditional application, chemical composition, and therapeutic effects of various medicinal materials in THSWD on breast cancer. In summary, we used network pharmacology to screen 58 main active ingredients from the 6 medicinal materials of THSWD, and constructed a network of “herbs-components-targets” (58 active ingredients and 386 protein targets) and “targets-pathway” interaction network (386 protein targets and 166 related key pathways). Screen the top 20 significantly enriched pathways, according to the number of related genes in the pathway, the PI3K/AKT, MAPK, MicroRNAs, FoxO, and HIF-1 signaling pathway genes are enriched with multiple targets. Among them, the PI3K/AKT and MAPK signaling pathways are closely related to the proliferation and metastasis of tumor cells, FoxO and HIF-1 signaling pathways can interfere with the tumor hypoxic microenvironment, and the MicroRNAs signaling pathway can regulate the EMT process of tumor cells and intervene in the process of tumor cell metastasis. It is revealed that the molecular mechanism of THSWD in the treatment of breast cancer is mainly by improving the microenvironment of tumor cells, regulating the process of tumor cell EMT, and inhibiting tumor cell proliferation and metastasis. It is predicted that baicalein, kaempferol, caffeic acid, amygdalin, quercetin, ferulic acid, gallic acid, catalpol, hydroxysafflor yellow A, paeoniflorin and other candidate effective ingredients in the treatment of breast cancer in THSWD are obtained. Application and development of new breast cancer treatment drugs provide reference. At present, we have initially explored the efficacy and mechanism of THSWD in inhibiting the growth and metastasis of HER-2 positive breast cancer through in network pharmacology. After that, based on the results of network pharmacology, we will further reveal its mechanism of action through in vivo efficacy tests.

## Data Availability

Not applicable.

## Abbreviations

ADME: Absorption, distribution, metabolis, mexcretion
AKT1: AKT Serine/Threonine Kinase 1
BS: Baishao, *Paeoniae Radix Alba*
CAF: CTX + ADM + 5-FU, Cyclophosphamide + Adriamycin + 5-fluorouridine
CCK-8: Cell Counting Kit-8
CX: Chuanxiong, *Chuanxiong Rhizoma*
DG: Danggui, *Angelicae Sinensis Radix*
DH: Dihuang, *Rehmanniae Radix*
EMT: Epithelial mesenchymal transition
FYN: FYN Proto-Oncogene, Src Family Tyrosine Kinase
GO: Gene Ontology
HER2: Human epidermalgrowth factor receptor-2
HH: Honghua, *Carthami Flos*
HRAS: HRas Proto-Oncogene, GTPase
IARC: The International Agency for Research on Cancer
KEGG: Kyoto Encyclopedia of Genes and Genomes
LVD: Lymph vessel density
MAPK1: Mitogen-Activated Protein Kinase 1
MAPK3: Mitogen-Activated Protein Kinase 3
MMP-9: Matrix metalloproteinase
MVD: Microvessel density
TCM: Traditional Chinese medicine
TR: Taoren, *Persicae Semen*
PIK3CA: Phosphatidylinositol-4,5-Bisphosphate 3-Kinase Catalytic Subunit Alpha
PIK3R1: Phosphoinositide-3-Kinase Regulatory Subunit 1
SRC: SRC Proto-Oncogene, Non-Receptor Tyrosine Kinase
STAT3: Signal Transducer And Activator Of Transcription 3
TCMSP: Traditional Chinese Medicine System Pharmacology database
THSWD: Taohong Siwu Decoction
WBC: White blood cell
